# A Case of Urgent Laryngectomy for Tracheoinnominate Fistula

**DOI:** 10.7759/cureus.94100

**Published:** 2025-10-08

**Authors:** Michael D Rockwell, Kaylee J Zettler, Charlotte S Taylor, Tejas M Maheshwari, Gina D Jefferson

**Affiliations:** 1 Otolaryngology-Head and Neck Surgery, University of Mississippi Medical Center, Jackson, USA; 2 Radiology, University of Mississippi Medical Center, Jackson, USA; 3 Pathology, University of Mississippi Medical Center, Jackson, USA

**Keywords:** bovine aortic arch, muscle flap, tracheoinnominate fistula, tracheotomy, vascular anomalies

## Abstract

Tracheotomy is a commonly performed otolaryngologic procedure utilized to provide a secure airway. Tracheoinnominate fistula (TIF) is a feared complication of tracheotomy. Thorough preoperative physical examination and review of anatomic neck imaging include the assessment of the great vessels' relationship to the trachea. Intraoperative assessment is also critical. We outline a complex case of TIF development after performing tracheotomy for a locoregionally advanced-stage supraglottic malignancy. The patient presented with acute airway obstruction necessitating urgent tracheostomy, and her anatomy was later recognized as consistent with a bovine aortic arch anomaly. She ultimately underwent urgent total laryngectomy. A literature review highlights the anatomical variations of aortic great vessels and various challenges that aberrant vascular anatomy poses to tracheostomy. We also describe the intraoperative findings, inpatient clinical course, multidisciplinary emergency management, and different preventive and repair options. The goal of this case report is to highlight the critical need for surgeons to carefully evaluate for vascular anomalies involving the great vessels and to provide potentially effective methods to prevent devastating complications.

## Introduction

The aorta forms during the third week of gestation and is formed from the six pairs of branchial arch arteries. The branchial arches begin to develop during the fourth and fifth weeks of gestation. During this process, the branchial arch arteries fuse together to form many arteries, including the brachiocephalic trunk (BT), left common carotid (LCC) artery, and left subclavian artery. The formation of these main branches of the aortic arch occurs during the fifth and sixth weeks of gestation. These great vessels typically arise from right to left on the arch of the ascending aorta. Malformations of the aortic arch can occur due to segments of branchial arch arteries persisting or regressing in error. There are eight recognized aortic arch variations. The type 1 variation describes the common arrangement of three great vessels arising from right to left on the aorta: the BT, LCC artery, and left subclavian artery. Type 2 refers to the bovine arch variation, the most common great vessel anomaly, in which the LCC artery has a common origin with the BT [1]. The term "bovine" refers to the resemblance of the innominate artery to a cattle head, but despite its name, it does not refer to the anatomical structure of the great vessels in cattle. A meta-analysis of 23,800 aortic arches found that 80% had the type 1 variation while 13% had a type 2 bovine arch arrangement. The bovine arch variation is further divided into type 1 and type 2 subtypes. The type 1 subtype is more common and represented by the BT and LCC artery origination from a shared trunk, and the LCC artery travels anterior to the trachea as far superiorly as the third tracheal ring. It is estimated that approximately 13% of the population have this vascular anomaly as our patient presented here [2].

Tracheotomy is usually performed with two primary approaches: the traditional open surgical tracheotomy (ST) and percutaneous dilatational tracheotomy (PDT) when deemed anatomically feasible. Regardless of the surgical approach, key anatomical landmarks for proper tube placement include the thyroid notch, cricoid cartilage, and sternal notch. The incision is typically made between the second and third tracheal rings. Placing the tube too far superiorly at the level of the cricoid cartilage risks erosion, cartilage fracture, or subglottic/tracheal stenosis. Conversely, inferior positioning below the third tracheal ring increases the likelihood of damaging mediastinal structures such as the innominate artery or later developing a tracheoinnominate fistula (TIF) [3]. There are instances, however, when it is preferable to create the tracheotomy between the first and second tracheal rings. In patients with locally advanced laryngeal cancer who will undergo total laryngectomy, an attempt to maximally preserve the trachea for the creation of a tension-free tracheostoma with optimal positioning for tracheoesophageal puncture is accomplished by tracheostomy placement between tracheal rings 1 and 2 [4].

A TIF is a rare but often fatal complication of tracheotomy [5]. This case report highlights the great vessel variant anatomy of a high-riding innominate artery and an aberrant bovine aortic arch resulting in a complicated post-tracheotomy course, the management of a TIF, and the impact on oncologic surgical planning.

## Case presentation

**Figure 1:**
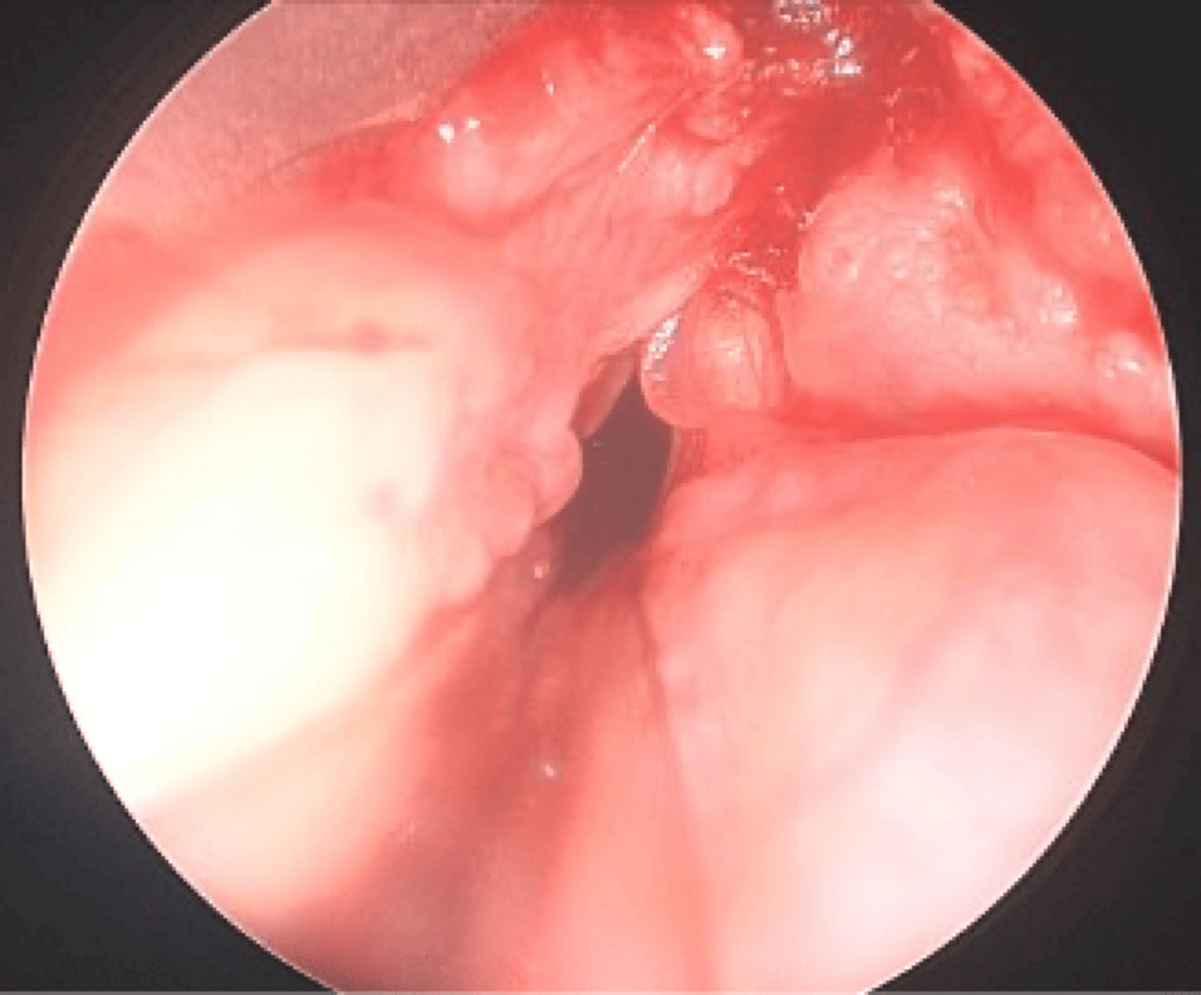
Right false vocal fold submucosal mass extending anteriorly into the paraglottic space

A 74-year-old woman with a history of group B chronic obstructive pulmonary disease (COPD) (2017 Global Initiative for Chronic Obstructive Lung Disease (GOLD) Classification) presented to the emergency department for two months of worsening dyspnea and hoarseness. Flexible laryngoscopy revealed an airway-obstructing right false vocal cord (FVC) mass and right vocal cord hypomobility (Figure 1). She underwent urgent tracheotomy and direct laryngoscopy with biopsy. A high-riding innominate artery overlying the third and fourth tracheal rings was palpated during the sterile preparation of the neck. During the performance of tracheotomy, visible pulsation of the high-riding innominate artery deep to soft tissue was observed inferiorly and was carefully avoided. Soft tissue was dissected free of the trachea, exposing the first three tracheal rings. A tracheotomy was then made between the second and third tracheal rings, and the tracheostomy tube was placed. Biopsies were taken of the right supraglottic mass and sent to pathology, which confirmed squamous cell carcinoma.

**Figure 2:**
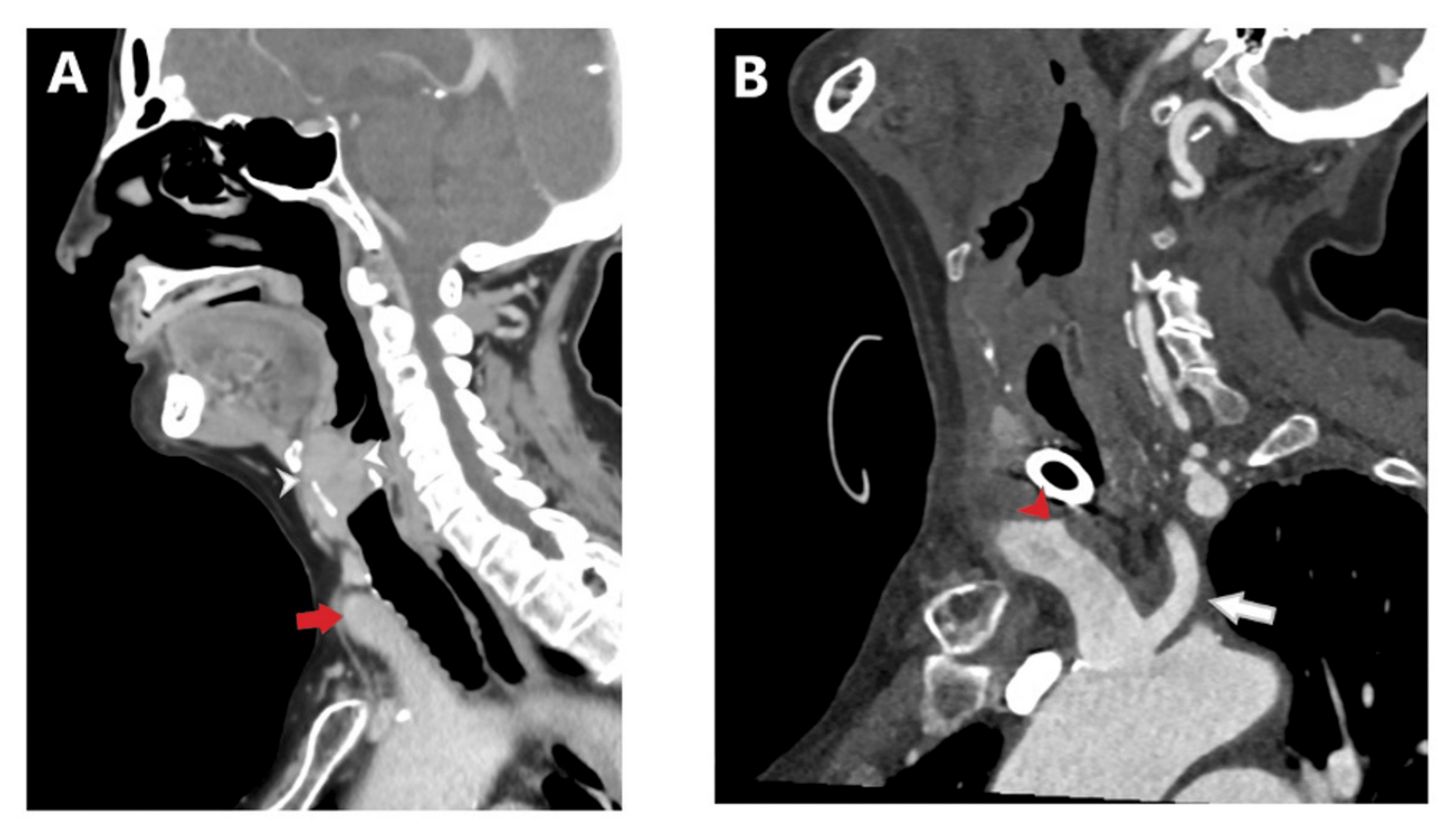
CT depiction of laryngeal cancer in relation to aberrant vascular anatomy (A) CT of the soft tissue neck with contrast in the sagittal plane shows a transglottic mass with extralaryngeal extension and effacement of the laryngeal airway (arrowheads). The innominate artery is high-riding, extending to the level of the first tracheal ring (red arrow). Note the smooth contour of the innominate artery as it contacts the trachea. This scan was obtained on 9/10/24 at an outside hospital preoperatively. (B) CT angiogram of the neck in an oblique sagittal plane performed post-tracheostomy shows a small triangular pseudoaneurysm along the posterior wall of the innominate artery (red arrowhead) with a sliver of contrast tracking to the tracheal wall, consistent with a TIF. The innominate artery and common carotid artery (white arrow) share a common origin, a "bovine arch" anatomic variant. CT: computed tomography; TIF: tracheoinnominate fistula

**Figure 3:**
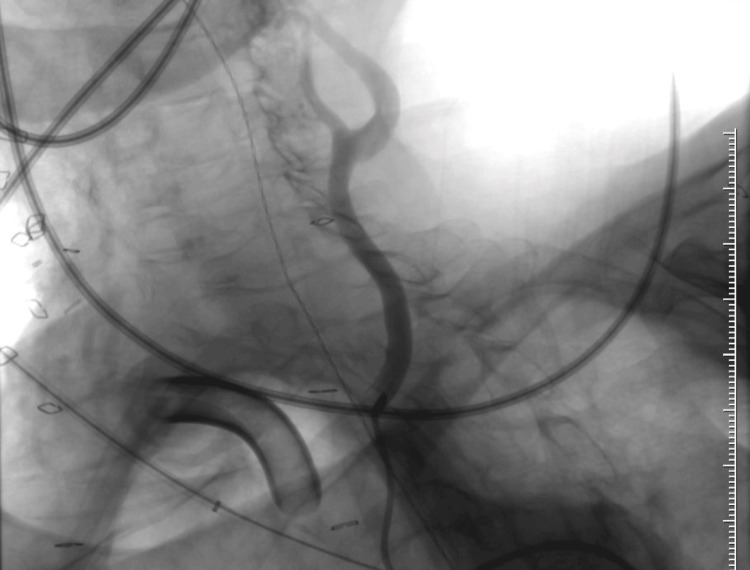
Intraoperative angiogram demonstrating the successful occlusion of the right subclavian artery with the patent left carotid artery

Routine cuffless tracheostomy tube change was performed on postoperative day (POD) 3 without issue. On POD 5, the patient experienced a high-volume bleed through the tracheostomy tube. Flexible tracheoscopy revealed a clot present on the distal tip of the tracheostomy tube with no active bleeding seen. The tracheostomy tube was replaced with a cuffed tube, and a computed tomography angiogram (CTA) of the head and neck revealed an area concerning for innominate artery exposure near the tracheostomy tube, increasing the risk of developing another bleed. A two-vessel aortic arch with a common origin of the LCC artery and BT was also noted (Figure 2A-2B). After consultation, vascular surgery performed an emergent innominate artery stent insertion, accessing the right common carotid artery (CCA) for the retrograde insertion of the stent. The abnormal origin of the LCC artery from the innominate artery precluded the ligation of the innominate artery. The patient experienced another high-volume pulsatile bleed from the tracheotomy requiring emergent repeat intervention by vascular surgery, who then performed a right CCA stent and innominate artery percutaneous transluminal angioplasty. This stent was deployed with enough overlap to stay within the previously placed innominate artery stent that preserved flow to the left CCA. Follow-up angiogram showed no further extravasation with the occlusion of the origin of the right subclavian artery containing the TIF segment (Figure 3). Postoperatively, the patient remained intubated and sedated under intensive care unit (ICU) care with no bleeding reported.

**Figure 4:**
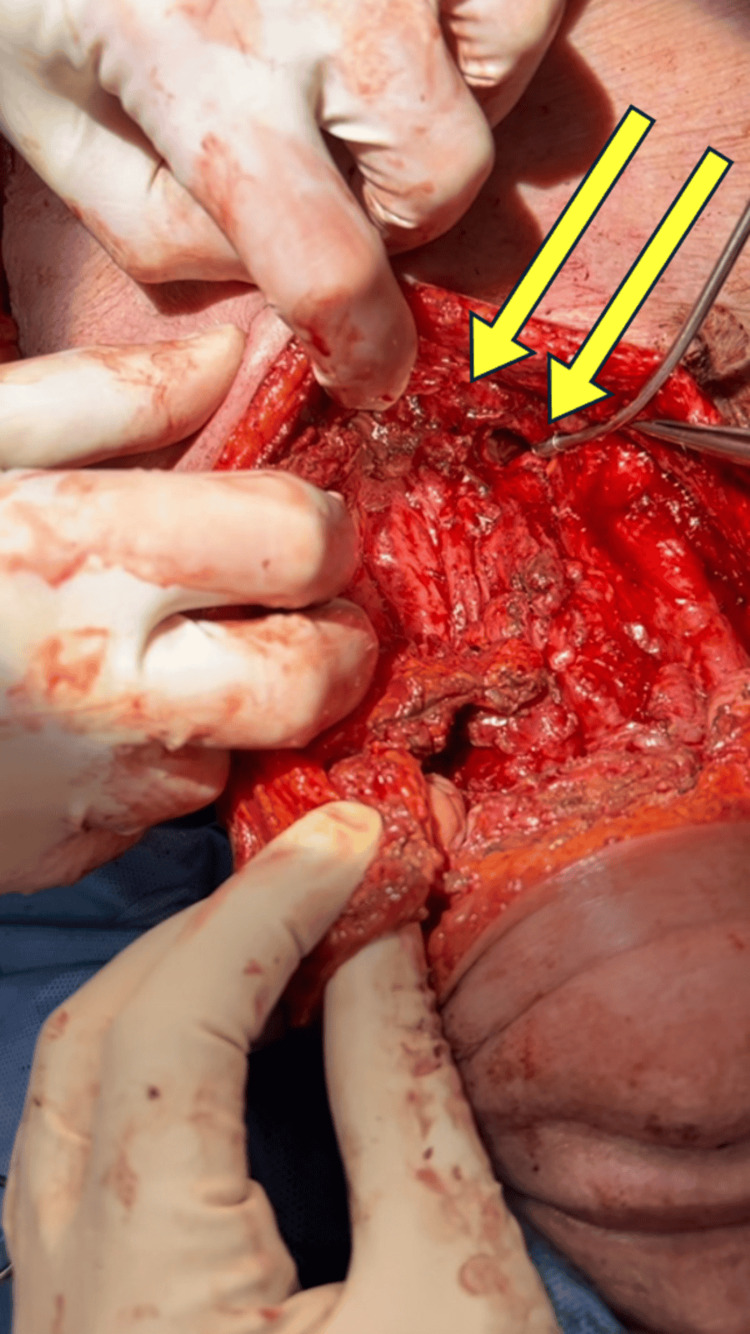
Depiction of the high-riding innominate artery and common carotid with intraluminal stent Yellow arrows indicate the innominate artery with intravascular stent crossing over the trachea.

**Figure 5:**
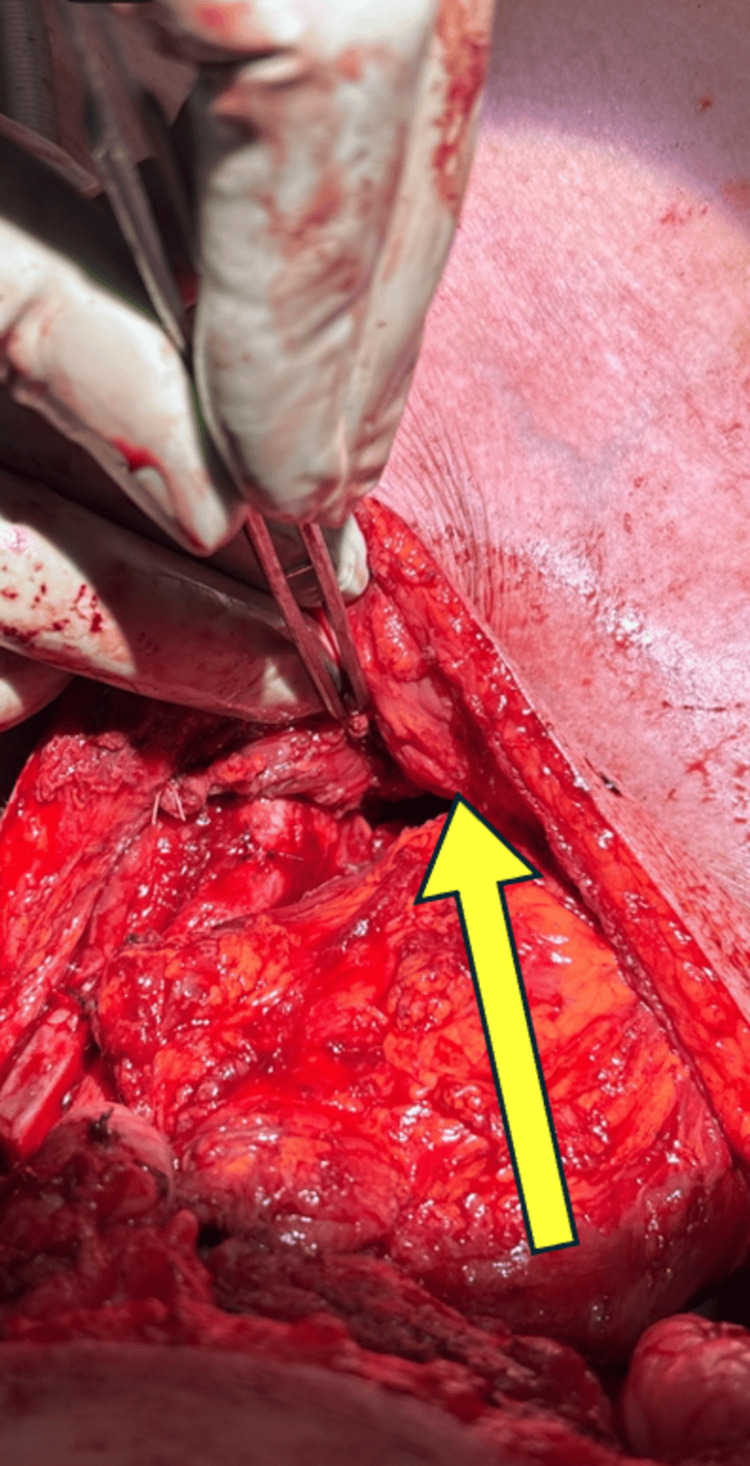
Interposition pectoralis muscle flap between the aberrant vasculature and tracheostoma Yellow arrow demonstrates the left pectoralis major muscle after split and rotation into place wrapped around the innominate artery to create a physical barrier between it and the tracheal wall.

Discussion at the multidisciplinary head and neck tumor conference confirmed the clinical American Joint Committee on Cancer (AJCC) stage of cT4a N2c M0 squamous cell carcinoma of the supraglottis originating from the right false vocal cord. After thorough discussion with the patient and family about the best oncologic outcomes in the setting of TIF, our collaborative decision was to proceed with urgent total laryngectomy, bilateral neck dissections, and left pectoralis major muscle flap for TIF repair and protection of the innominate artery. Surgery was uneventful, and the left pectoralis major muscle was split and secured to the pre-sternal fascia and tucked between the innominate artery and tracheostoma to protect the vessel (Figures 4-5). Utilization of the left pectoralis muscle was secondary to a prior open vascular procedure attempting to mitigate the TIF. Our team was uncertain of the remaining vascular supply to the right pectoralis major muscle after open exposure by vascular surgery of both the carotid artery and innominate artery. A decision was made to maintain the patient in the hospital until the planned initiation of the oral diet on POD 11. After an esophagogram showed no evidence of neopharyngeal leakage, the patient began an oral diet.

**Figure 6:**
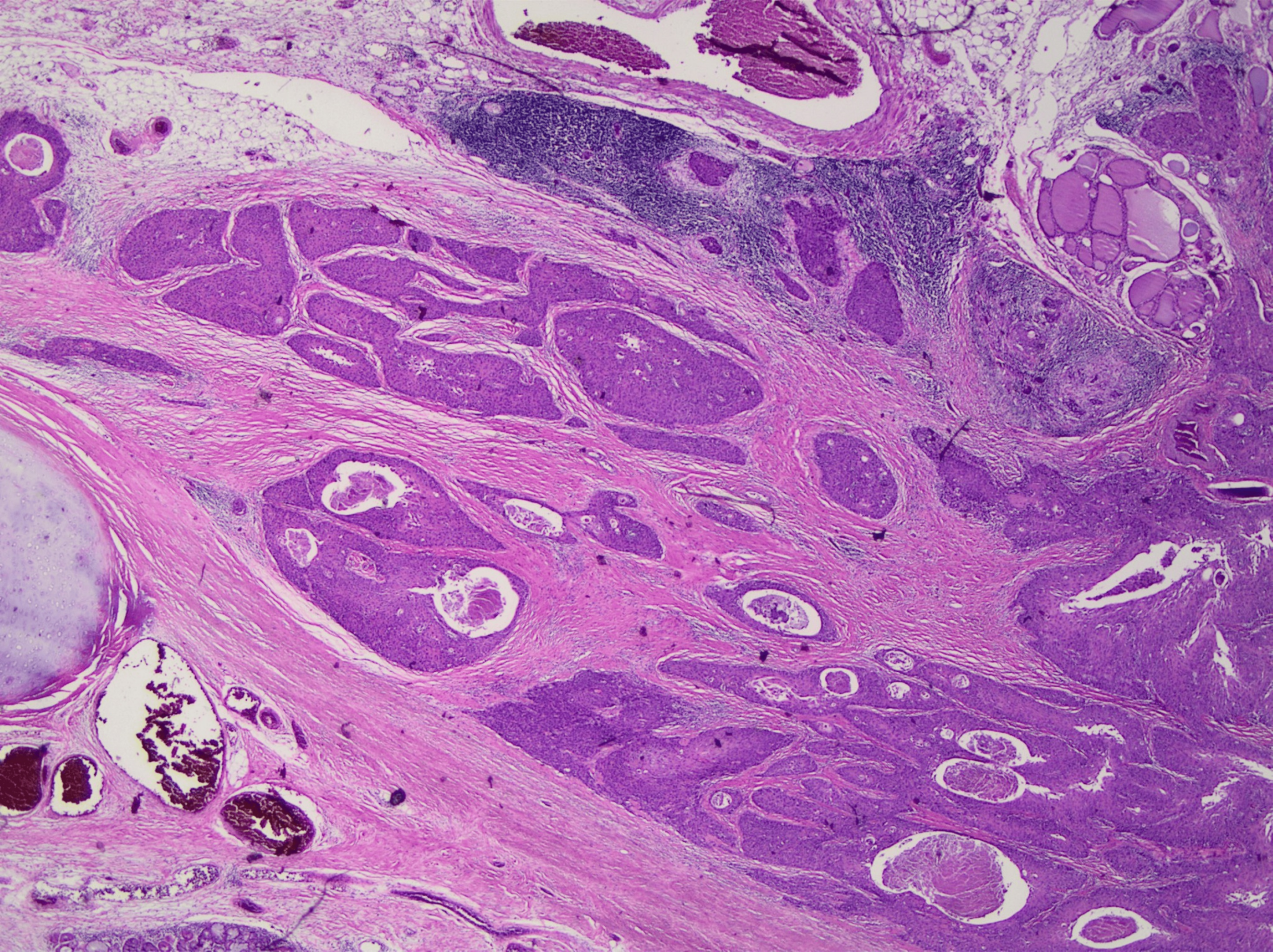
Squamous cell carcinoma extending beyond the thyroid cartilage and invading the thyroid parenchyma Hematoxylin and eosin stain, low magnification.

**Figure 7:**
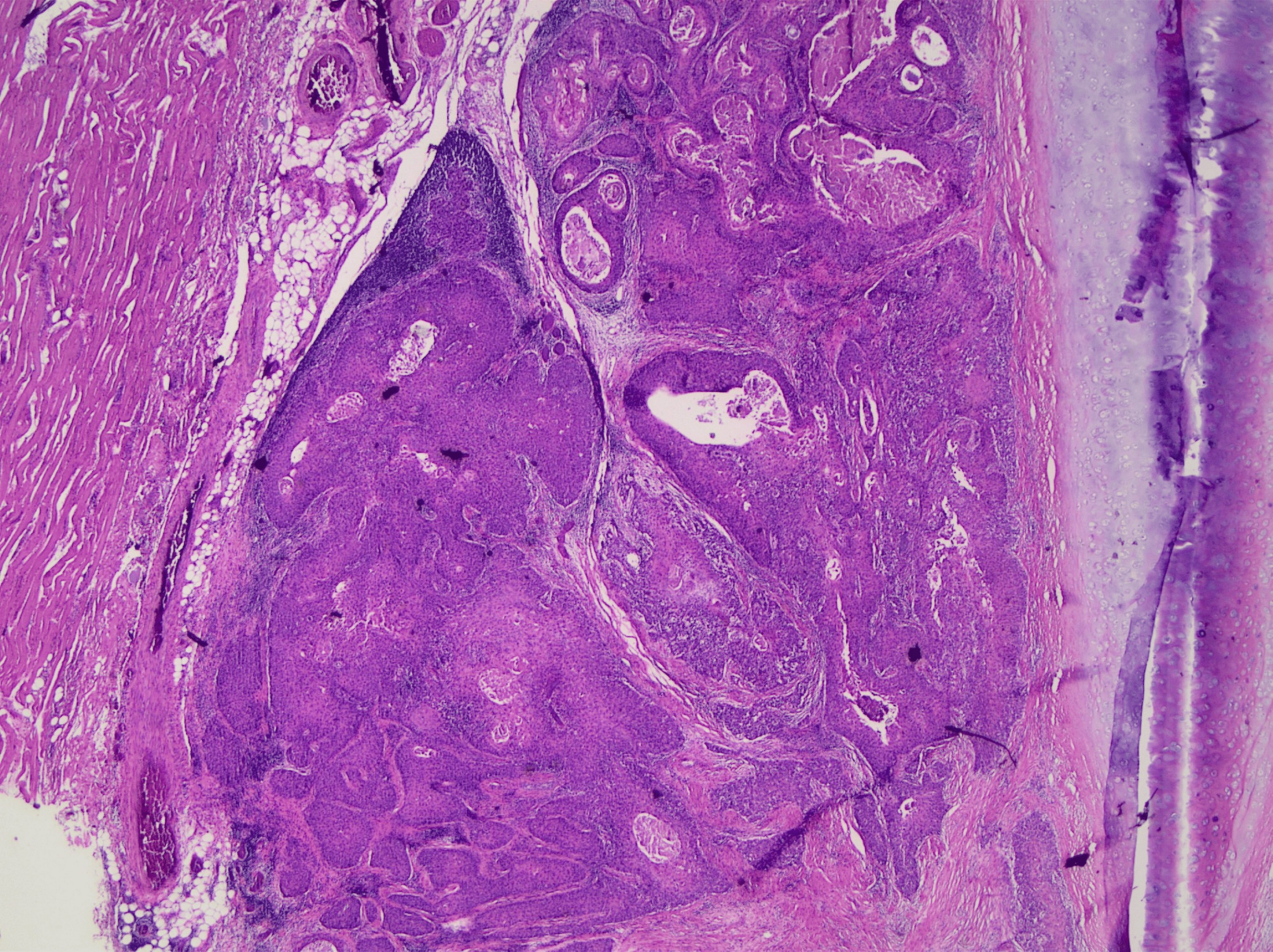
Squamous cell carcinoma invading the strap muscles Hematoxylin and eosin stain, low magnification.

**Figure 8:**
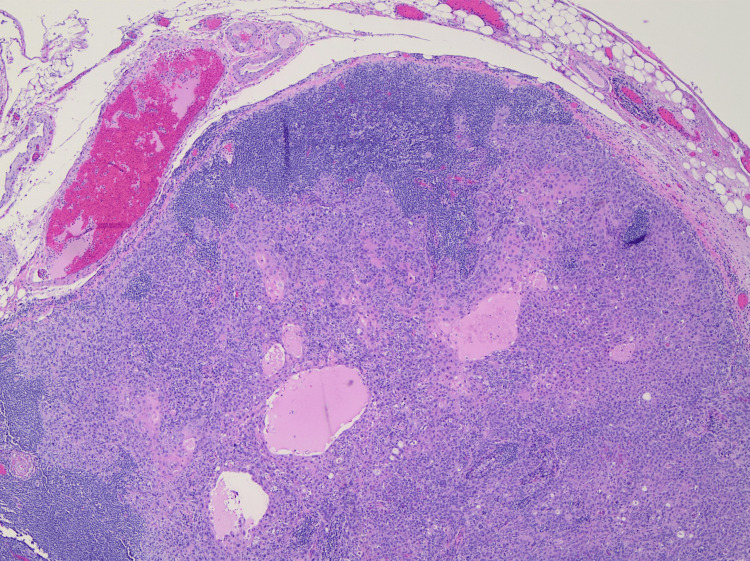
Squamous cell carcinoma metastatic to the lymph node Hematoxylin and eosin stain, low magnification.

The final surgical pathology resulted in a pT4a N1 disease of the supraglottis with extralaryngeal soft tissue involvement with perineural invasion identified (Figures 6-8). Recommendations were made for adjuvant chemoradiation therapy. Due to social constraints, the patient was transferred to the oncology service for inpatient adjuvant treatment. Prior to treatment, recommendations were made for gastrostomy tube placement given the lack of significant diet advancement. On the morning of POD 18, a laparoscopic gastrostomy tube placement was performed, where tracheostomal intubation was accomplished uneventfully using a reinforced endotracheal tube. During recovery, there was an acute recurrent peristomal bleed, and the Utley maneuver was performed with a temporary tamponade achieved. After a multidisciplinary discussion, it was decided that further vascular intervention was likely futile, as the only life-saving interventions available would result in massive stroke and continued high mortality risk. This was discussed with her family. She was then transitioned to comfort care and subsequently passed away.

## Discussion

Although roughly 13% of aortic arches possess the bovine aortic arch variant, very few cases of a TIF occurring in the context of a high-riding innominate artery in stage 4 laryngeal malignancy are described in the literature, with none reporting the need for urgent total laryngectomy [6,7]. Our patient's presentation and experience provide an opportunity to review airway management for patients in need of tracheotomy with aberrant great vessels in proximity to the airway. Urgent laryngectomy in our case was deemed necessary to allow the removal of the rigid tracheostomy tube and insertion of a silastic laryngectomy tube to minimize further ulceration and reformation of great vessel fistulization.

First, patients requiring a surgical airway prior to the definitive treatment of their laryngeal cancer are not typically good candidates for chemoradiation with significantly decreased overall survival [8-10]. Given that total laryngectomy will provide the basis of definitive treatment, it is advisable to retain as much of the tracheal length as possible when performing tracheostomy. Ideally, the tracheotomy placement, when oncologically possible, is best placed between rings 1 and 2. Our patient did not have a significant infraglottic extension of disease and, therefore, could have undergone tracheostomy placement more proximally.

**Figure 9:**
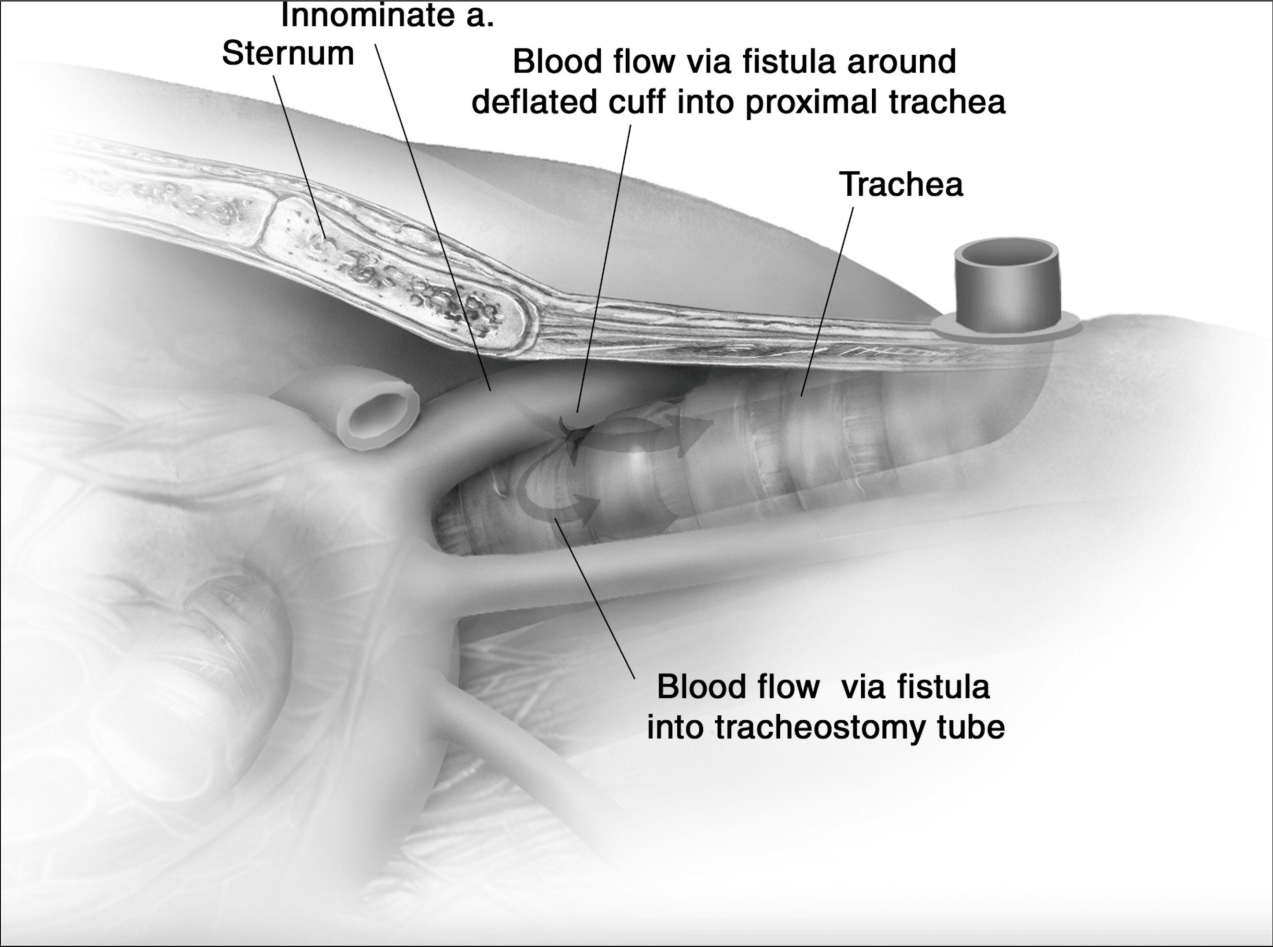
Visual depiction of tracheoinnominate fistula formation

Second, it is important to consider means to prevent the formation of TIF when encountering a high-riding vascular structure during tracheostomy. TIF results from pressure-induced ischemia from the tracheostomy tube, leading to mucosal breakdown and the subsequent erosion of the anterior tracheal wall into the posterior wall of the innominate artery (Figure 9). The incidence of TIFs ranges from 0.1% to 1%, with a high mortality rate of 65-73% [5,11]. Survival depends largely on the timing of surgical intervention, but even those who undergo corrective surgery face a high risk of intra- and postoperative complications, including infection. TIFs can develop as early as three days or as late as several years after tracheostomy, with an average onset of 29 days [5,12]. A sentinel bleed around the tracheostomy site is often an early warning sign, and pulsatile bleeding from the stoma or tracheostomy tube lumen indicates likely fistula formation. Prompt recognition is critical, and a CTA scan can help identify the fistula site for urgent operative repair, whether via median sternotomy with innominate artery ligation or via endovascular stenting. Although the innominate artery often crosses anterior to the ninth tracheal ring, it can commonly cross anterior to the sixth to 13th rings. There are reports of aberrant variations that cross the trachea as far superiorly as the second to fourth tracheal rings, where a tracheotomy is usually performed [13]. This can lead to catastrophic hemorrhage if not assessed thoroughly in the pre- or perioperative settings. Creation of a physical barrier can prevent TIF formation.

Netzer et al. performed an inferiorly based inverted U-shaped tracheal wall flap on a series of two patients with high-riding innominate arteries discovered by palpation and preoperative neck ultrasound. This was created on the anterior tracheal wall from the second and third rings, and the flap was draped over the innominate artery and sutured to the lower aspect of the skin incision [13]. This allowed for physical protection against erosion from the concave portion of the tracheotomy tube, and no bleeding from the site was reported 46 months postoperatively. A more robust physical barrier, a muscle flap, has been performed since the 1970s, as first described by Dellon et al., who performed a rotational sternocleidomastoid muscle (SCM) flap and rotational superior and inferior skin pedicle flaps to create a permanent tracheotomy [14]. Additional methods include using the thymic tissue as bulk to separate the trachea and innominate artery [15]. Chuang et al. reported performing a rotational SCM flap to provide further tissue protection after an iatrogenic TIF was created after a tracheostoma dilation in a patient with a history of total laryngectomy [16]. For small TIFs, a two-layer investing suture for closure may be adequate. However, for medium fistulas ranging between 10 and 20 mm in length, success rates are higher when a two-layer closure is performed in addition to a strap muscle rotational flap. In our case, we performed a pectoralis major muscle rotational flap to create separation between the innominate artery and the anterior tracheal wall. Therefore, a variety of muscle and tracheal flaps are available depending on the size of the fistula.

Third, it is important to note additional methods of managing a TIF. Our on-call team did not place a more proximal tracheostomy, and this resulted in TIF secondary to the aberrant vascular anatomy. The traditional surgical approach for TIF is a median sternotomy with innominate artery ligation; however, patients undergoing this procedure face an increased risk of local and sternal wound infections. For high-risk surgical candidates, such as our patient with limited pulmonary reserve from group B COPD, endovascular repair is a less invasive and less morbid alternative. Additionally, endovascular stents are used to repair various causes of innominate artery injury, including treating innominate artery pseudoaneurysms from central venous access and in the trauma setting. Two case series found no significant difference in survival rates between open and endovascular approaches, both averaging 48-49% [11,12]. Endovascular methods were also associated with fewer postoperative complications, 30%, compared to 50% with sternotomy [12]. In our patient, the innominate artery could not be ligated due to its common trunk with the left CCA, causing concern for occluding both the right and left CCAs. A second procedure, right CCA stent and innominate artery percutaneous transluminal angioplasty, was performed after the patient's second large tracheostomy bleed with no leakage of contrast afterward.

Although associated with increased morbidity as discussed previously, open sternotomy and innominate artery ligation provide a more definitive solution. Although not routinely performed in the present day, Tokita et al. have described prophylactic innominate artery resection to reduce the risk of TIF formation in patients possessing a high-riding innominate artery and at high risk for TIF. The authors performed innominate artery ligation in an elective manner, followed by tracheotomy six weeks later. However, the authors did report that the risk of stroke is higher if the ligation were performed on an emergent basis prior to tracheotomy [17]. Additional considerations include the need for either preoperative MR brain angiography to assess for incomplete circle of Willis or intraoperative electroencephalogram (EEG) to assess for changes indicative of stroke after the innominate artery is clamped prior to ligation. Vascular surgeons must also be prepared to perform extra-anatomic bypass, such as axillofemoral bypass pending intraoperative EEG findings. All these procedures are precluded for patients in need of urgent airway intervention.

Finally, direct communication between the anesthesia team and the surgical airway team allows discussion of the safest approach to secure the airway. Despite the anesthesia and general surgery teams' awareness of our patient's increased risk of innominate artery bleeding, otolaryngology was not present for the stomal intubation and attempted extubation during the gastrostomy tube placement. Our rotational muscle flap repair had lasted 18 days at this juncture and had the potential to last for a longer duration. Although the patient may have had the same outcome if the otolaryngology team familiar with her anatomy was present, there was a missed opportunity for careful guidance during intubation to reduce the risk of bleeding.

## Conclusions

Although tracheotomy is a frequently performed and life-saving procedure, it is critical for providers to perform thorough physical examinations and obtain appropriate imaging to detect a high-riding innominate artery or aberrant vasculature. Innominate injury can lead to catastrophic hemorrhage and significant morbidity, particularly death. In a non-emergent setting, if a high-riding innominate artery or aberrant vascular pattern is identified preoperatively, thereby placing the patient at a high risk of TIF, prophylactic innominate artery ligation near the planned tracheotomy site can be considered, with consideration for possible stroke as a risk. Reviewing available preoperative imaging for such vascular anomalies is also a critical tool in determining the surgical approach. If encountered intraoperatively at the time of tracheostomy, a rotational muscle flap or placement of thymic tissue to create an interposition between the innominate artery and anterior tracheal wall can also mitigate the risk of fistula formation. Such cautionary measures will ensure greater success in safely approaching procedures involving the anterior neck.
